# Social, behavioral and environmental correlates of tobacco use among adults in a tribal population in northern India

**DOI:** 10.3389/fpubh.2026.1927436

**Published:** 2026-10-08

**Authors:** U. Venkatesh, Arshad Ahmed, R. Durga, Anand Mohan Dixit, Vibha Dutta

**Affiliations:** 1 Department of Community & Family Medicine, All India Institute of Medical Sciences, Gorakhpur, Uttar Pradesh, India; 2 Independent Researcher, Gorakhpur, Uttar Pradesh, India; 3 All India Institute of Medical Sciences, Gorakhpur, Uttar Pradesh, India

**Keywords:** India, social determinants of health, tobacco use, tribal populations, workplace smoking exposure

## Abstract

**Background:**

Tobacco use remains a leading preventable cause of morbidity and mortality worldwide and disproportionately affects socially and economically marginalized populations. Although national surveys provide overall prevalence estimates, evidence on the socio-demographic, behavioral, and environmental correlates of tobacco use in underserved tribal communities remains limited. This study examined the prevalence and correlates of tobacco use among adults residing in tribal villages in northern India.

**Methods:**

A community-based cross-sectional study was conducted among adults aged ≥20 years residing in five tribal villages of Gorakhpur district, northern India. A complete household enumeration was undertaken between March and June 2024. Data were collected using a structured questionnaire covering socio-demographic characteristics, alcohol consumption, household smoking exposure, and workplace smoking exposure. Current tobacco use (smoked or smokeless) was self-reported. Associations between explanatory variables and tobacco use were assessed using chi-square tests and multivariable logistic regression to estimate adjusted odds ratios (AORs) with 95% confidence intervals (CIs). Model calibration was assessed using the Hosmer–Lemeshow goodness-of-fit test and receiver operating characteristic (ROC) curve analysis was conducted as a supplementary exploratory assessment of model discrimination.

**Results:**

A total of 2,236 adults participated in the study. The overall prevalence of current tobacco use was 10.7%. Tobacco use was significantly higher among men than women (17.9% vs. 2.4%; *p* < 0.001) and increased with advancing age. After adjustment, male sex (AOR 2.12, 95% CI 1.08–4.18), older age (≥60 years: AOR 3.42, 95% CI 1.67–6.99), and workplace smoking exposure (AOR 3.19, 95% CI 1.70–6.00) remained independently associated with tobacco use. Although occupational category and household smoking exposure were significant in unadjusted analyses, these associations were attenuated after multivariable adjustment. The final model demonstrated good calibration (Hosmer–Lemeshow *p* = 0.059) and high apparent discriminatory performance (AUC 0.93, 95% CI 0.91–0.95).

**Conclusion:**

Tobacco use in this tribal population was associated with gender, older age, and workplace smoking exposure. The independent association between workplace smoking exposure and tobacco use supports consideration of strengthened smoke-free workplace policies, particularly within informal labor settings. Integrating tobacco-control interventions with community-based behavioral risk reduction strategies through primary healthcare systems may help reduce tobacco-related disparities in underserved tribal populations.

## Introduction

Tobacco use remains one of the leading preventable causes of morbidity and premature mortality worldwide, accounting for more than seven million deaths annually and substantial losses in life expectancy and economic productivity (1–3). Despite sustained global tobacco control efforts over the past two decades, tobacco consumption continues to pose a major public health challenge. Recent estimates from the World Health Organization (WHO) indicate that although the global number of tobacco users declined from approximately 1.38 billion in 2000 to about 1.2 billion in 2024, nearly one in five adults worldwide still uses tobacco, highlighting persistent inequalities in tobacco exposure and tobacco-related disease burden (4–6).

Global progress in tobacco control has been driven largely by the implementation of the WHO Framework Convention on Tobacco Control (WHO FCTC) and the MPOWER policy package, which emphasize surveillance, protection from second-hand smoke exposure, cessation support, health warnings, advertising restriction, and taxation (7–9). Evidence suggests that comprehensive implementation of these measures has contributed to decline in tobacco use prevalence and smoking-attributable mortality in several regions (10–12). However, progress has been uneven across populations and geographical contexts. While reductions in tobacco use have been observed in many high-income settings, the burden remains disproportionately concentrated in low- and middle-income countries and among socially disadvantaged population groups (13, 14).

South-East Asia continues to account for a substantial share of global tobacco consumption due to its large population size, widespread availability of smoked and smokeless tobacco products, and persistent socio-economic inequalities (15–17). In India, tobacco use represents a particularly complex public health challenge. Multiple forms of tobacco consumption coexist within diverse cultural and occupational settings, and marked disparities by gender, education, and socio-economic status contribute to an uneven distribution of tobacco-related health risks (18, 19). Tobacco-attributable mortality in India exceeds one million deaths annually and contributes substantially to the national burden of non-communicable diseases, tuberculosis, and oral cancers (1, 20).

Evidence from nationally representative surveys, including the Global Adult Tobacco Survey (GATS), consistently demonstrates that tobacco use in India is strongly socially patterned rather than randomly distributed across the population (18, 19). Higher prevalence has been observed among men, individuals with lower educational attainment, and populations engaged in manual or informal occupations (21–25). These patterns are consistent with social epidemiological frameworks, which emphasize the importance of broader structural, occupational, and environmental contexts in understanding health behaviors rather than viewing them solely as matters of individual choice (26–28). In particular, social disadvantage, workplace norms, and smoking exposure may be relevant to tobacco use patterns within specific communities.

Studies from South Asia further highlight the clustering of behavioral risk factors within vulnerable populations. Empirical analyses from Nepal and neighboring countries have demonstrated strong associations between tobacco use, older age, lower education, alcohol consumption, and socio-economic disadvantage (29–31). Such clustering reflects broader social and environmental determinants of health and suggests that tobacco use often co-occurs with other behavioral risk factors within shared social contexts (32–34). Understanding these interconnected patterns is therefore important for designing effective and context-sensitive tobacco control strategies.

Despite substantial evidence on the social factors associated with tobacco use at the national level, important gaps remain in understanding how structural disadvantage, behavioral risk clustering household smoking exposure, and workplace smoking exposure are associated with tobacco use patterns. In India, tribal communities remain underrepresented in epidemiological research and national health surveillance systems, despite facing persistent socio-economic vulnerability and limited access to health resources (35). Moreover, relatively few studies have jointly examined the role of behavioral factors such as alcohol consumption alongside environmental exposures such as household and workplace smoking environments in relation to tobacco use patterns within such communities (36, 37).

Addressing these gaps requires analytical approaches that move beyond purely descriptive assessments of individual risk factors and instead examine how social, behavioral, and environmental factors are associated with tobacco use behaviors. In particular, workplace smoking exposure may be associated with tobacco use in informal labor settings where smoke-free regulations are weakly enforced and tobacco consumption may be commonly observed within social environments.

In this context, the present study examines the socio-demographic, behavioral, and environmental correlates of current tobacco use among adults aged 20 years and above in a marginalized tribal population in northern India. Using individual-level data and a multivariable analytical framework, this study aims to examine the association of structural socioeconomic conditions, behavioral risk clustering, household smoking exposure and workplace smoking exposure with tobacco use patterns within a historically marginalized community. By identifying population subgroups with higher prevalence of tobacco use and contextual factors associated with tobacco use, the study seeks to generate evidence that can inform more targeted and equity-oriented tobacco control strategies in alignment with WHO FCTC objectives and Sustainable Development Goal target 3.a (38, 39).

## Methods

### Study setting

The study was conducted in five tribal villages located in the Gorakhpur sub-division of Uttar Pradesh, India. The area lies between 83°23′30″-83°29′15″E longitude and 26°40′15″-26°48′15″N latitude (Figure 1). The region is characterized by flat alluvial terrain and a monsoon climate. According to the Census of India (2011), the wider Gorakhpur district has a population of 4.44 million, of whom tribal communities constitute about 0.4% of the total population (40).

**Figure 1:**
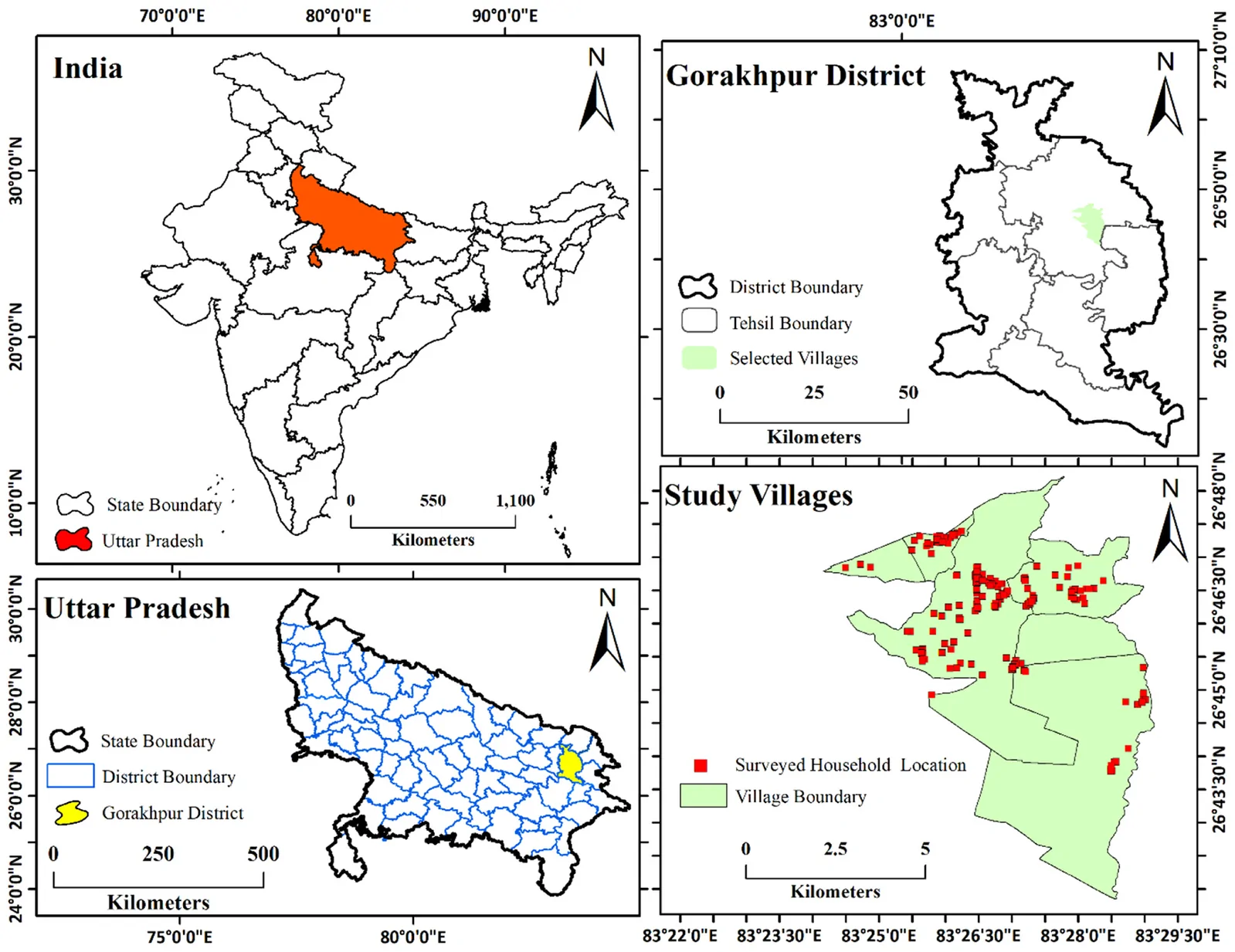
Location of the study area, Gorakhpur, India.

### Study population

The study population comprised members of the “*Vantangiya*” tribal community, a historically migrant population group residing in the selected villages. This community was originally resettled in the region during the early twentieth century to support afforestation activities on government land. The term “*Vantangiya*,” derived from the Hindi word *van* (forest), reflects the community's historical association with forest-based labor and livelihoods.

### Study design

A community-based cross-sectional study design was adopted to examine socio-demographic, behavioral and environmental correlates of tobacco use among adults residing in the selected villages (18, 19).

### Data collection

A complete household enumeration of all households in the five selected villages was conducted between March and June 2024. Individuals aged 18 years and above were eligible for the household survey and were enumerated during the field survey. However, the present analysis was restricted *a priori* to adults aged 20 years and above to maintain a consistent analytical age range for the assessment of tobacco-use patterns and their associated factors. Accordingly, participants aged 18–19 years were not included in the present analysis. A total of 4,000 individuals aged 18 years and above were enumerated and interviewed using a structured questionnaire. After applying the prespecified analytical age criterion, 1,764 participants aged 18–19 years were excluded, resulting in a final analytical dataset of 2,236 adults aged 20 years and above.

### Survey tools and procedures

Data were collected using a structured questionnaire comprising three domains: (i) sociodemographic characteristics, including age, gender, marital status, education, occupation, and household income; (ii) behavioral factors, including tobacco use and alcohol consumption; and (iii) environmental exposures, including household smoking exposure and workplace smoking exposure. The questionnaire was administered to eligible participants during the household survey by the study team. Tobacco use was assessed through self-reported current use of tobacco products at the time of the survey (18, 19). The assessment included both smoked and smokeless forms of tobacco. Participants were classified as current tobacco users if they reported current use of any smoked or smokeless tobacco product, whereas those who did not report current use were classified as non-users. For the statistical analysis, tobacco use was coded as a binary outcome (0 = non-user; 1 = current tobacco user).

The questionnaire also collected information on alcohol consumption, household smoking exposure, and workplace smoking exposure. Household smoking exposure was defined as the presence of at least one other household member who smoked tobacco (32, 36). Workplace smoking exposure was assessed among participants who reported attending a workplace where smoking occurred (21, 23). All exposure and behavioral variables were based on participant self-report (21, 23).

### Outcome variable

The primary outcome variable was current tobacco use. Current tobacco use was based on self-reported use at the time of the survey and included both smoked and smokeless tobacco products. Participants were classified as current tobacco users if they reported using any smoked or smokeless tobacco product at the time of the survey. Individuals who did not report current use were classified as non-users. For analytical purposes, tobacco use was coded as a binary variable: (0 = non-user, and 1 = current tobacco user). Binary logistic regression models were used to examine associations between explanatory variables and current tobacco use.

Because participants were recruited within households and villages, the potential for within-household and within-village correlation was considered in the interpretation of the regression findings. Standard binary logistic regression was used to estimate individual-level associations between the measured explanatory variables and current tobacco use. Household- and village-level clustering was not explicitly incorporated through multilevel random effects or cluster-adjusted standard errors. The regression model adjusted for the measured individual-level sociodemographic, behavioral, and exposure variables included in the analysis. Nevertheless, conventional logistic regression assumes independence of observations, and residual correlation arising from shared household or village characteristics may affect the precision of the estimated associations. Therefore, the regression estimates are interpreted with this potential clustering in mind, and the absence of explicit cluster-level adjustment is acknowledged as a limitation of the present analysis.

### Explanatory variables

Based on existing epidemiological literature on tobacco use and behavioral risk factors in adult populations, a set of sociodemographic, behavioral and environmental exposure variables was selected for analysis (18, 19, 26). Marital status and household income were collected as part of the socio-demographic assessment and are presented descriptively in Table 1. However, these variables were not included in the regression analyses because they were not prespecified as core explanatory variables in the study's conceptual and analytical framework. The multivariable model was restricted to variables considered directly relevant to the study objectives and supported by prior epidemiological evidence, including age, gender, education, occupation, alcohol consumption, and household and workplace smoking exposure. Marital status and household income were therefore retained for descriptive characterization rather than multivariable adjustment. Sociodemographic variables included: age group (20–29, 30–59, ≥60 years); gender (male, female); education level. Education was originally recorded in multiple categories and subsequently recoded into three groups (illiterate, primary education and secondary education or above) to improve analytical stability. Socioeconomic characteristics were captured through occupation, which was recoded into five categories: homemaker, labor/farmer, student, unemployed, other occupations. Behavioral factors included alcohol consumption, recorded as a self-reported binary variable (yes/no). Environmental exposure variables included household smoking exposure, defined as the presence of any other household member who smoked tobacco, and workplace smoking exposure, defined as exposure to smoking in the workplace among participants who reported attending a workplace. All explanatory variables were treated as categorical variables in the analysis (see details in Table 2).

**Table 1:** Socio-demographic characteristics of the study population (*N* = 2,236).

Characteristic	*n* (%) or Mean ±SD
Age (years)	**41.1** **±14.7**
Age group (years)	
20–29	512 (22.9)
30–59	1,293 (57.8)
≥60	431 (19.3)
Gender	
Male	1,197 (53.5)
Female	1,039 (46.5)
Marital status	
Married	1,809 (80.9)
Unmarried	355 (15.9)
Separated/Widowed	72 (3.2)
Education level	
Illiterate	1,411 (63.1)
Primary (1st−5th)	431 (19.3)
Secondary and above	394 (17.6)
Occupation	
Homemaker	756 (33.8)
Labor/Farmer	865 (38.7)
Student	113 (5.1)
Unemployed	292 (13.1)
Other occupations†	210 (9.4)
Monthly household income (INR)	**3,122** **±3,477**

†Other occupations include business owners, drivers, private sector workers, vendors and skilled workers. Values are presented as *n* (%) unless otherwise specified. Percentages may not total 100 due to rounding. Bold values indicate continuous variables presented as mean ± SD.

**Table 2:** Variable coding and reference categories used in regression analyses.

Variable	Original categories (as collected)	Recoded categories (used in analysis)	Coding	Reference category
Tobacco use	Non-user, Current tobacco user	0 = No, 1 = Yes	Binary	No
Age group (years)	20–29, 30–59, ≥60	20–29, 30–59, ≥60	Dummy	20–29
Gender	1 = Male, 2 = Female	1 = Male, 2 = Female	Dummy	Female
Education level	1 = Illiterate, 2 = Primary, 3 = Secondary, 4 = Higher secondary, 5 = Graduate, 6 = post-graduation	1 = Illiterate; 2 = Primary; 3 = Secondary+	Dummy	Illiterate
Occupation	1 = Homemaker; 2 = Labor/Farmer; 3 = Student; 4 = Unemployed; 5 = Other occupations	1 = Homemaker; 2 = Labor/Farmer; 3 = Student; 4 = Unemployed; 5 = Other occupations	Dummy	Other occupations
Alcohol consumption	0 = No, 1 = Yes	0 = No, 1 = Yes	Dummy	No
Household smoking exposure	0 = No, 1 = Yes	0 = No, 1 = Yes	Dummy	No
Workplace smoking exposure	0 = No, 1 = Yes	0 = No, 1 = Yes	Dummy	No

Education and occupation variables were collapsed to reduce sparse cells and improve model stability. Dummy coding was applied for all categorical variables.

### Statistical analysis

Descriptive statistics were used to summarize the socio-demographic characteristics of the study population. Continuous variables are presented as mean ± standard deviation (SD), while categorical variables are presented as frequencies and percentages (41).

### Bivariate analysis

Bivariate associations between current tobacco use and explanatory variables were assessed using Pearson's chi-square test. Variables examined included age group, gender, education level, occupation group, alcohol consumption, household smoking exposure and workplace smoking exposure. These variables were selected based on prior epidemiological evidence on the factors associated with tobacco use and were examined to identify potential associations with the outcome variable.

### Logistic regression analysis

Binary logistic regression analysis was performed to examine factors associated with current tobacco use. Initially, univariate logistic regression models were fitted to estimate crude odds ratios (ORs) with 95% confidence intervals (CIs) for each explanatory variable. Subsequently, a multivariable logistic regression model was constructed in which all selected socio-demographic, behavioral and environmental exposure variables were entered simultaneously. This approach allowed estimation of adjusted odds ratios (AORs) while accounting for potential confounding by the measured covariates included in the model. The final multivariable model included the following prespecified variables: age group, gender, education level, occupation group, alcohol consumption, household smoking exposure, and workplace smoking exposure. Marital status and household income were not included in the regression models because they were collected for descriptive characterization but were not prespecified as core explanatory variables in the analytical framework. These variables were selected *a priori* based on existing epidemiological evidence on the social factors associated with tobacco use and behavioral risk factors in adult populations (18, 19, 21, 26, 28), as well as their relevance to the conceptual framework of social and environmental factors associated with health.

### Model diagnostics and validation

Model calibration was evaluated using the Hosmer–Lemeshow goodness-of-fit test (Table 3). Because the primary objective of the study was to identify factors associated with current tobacco use rather than to develop a clinical or population-level prediction tool, receiver operating characteristic (ROC) curve analysis was conducted as a supplementary exploratory assessment of the discriminatory performance of the fully adjusted multivariable logistic regression model. The ROC curve was generated using predicted probabilities from the final model, and the area under the curve (AUC) with 95% confidence intervals was calculated. An exploratory probability threshold was identified using the Youden index to describe the sensitivity and specificity of the model within the study sample. This threshold was not intended to represent a clinically validated or universally applicable screening cut-off. The sensitivity, specificity, and Youden index were reported only to provide a descriptive summary of the trade-off between sensitivity and specificity at an exploratory probability threshold within the study sample. These measures were not intended to establish diagnostic accuracy, clinical utility, or a population-level screening threshold.

**Table 3:** Model fit assessment using Hosmer–Lemeshow test.

Model	χ^2^	df	*p*-value	Interpretation
Multivariable logistic regression	15.01	8	0.059	Adequate model fit

Hosmer–Lemeshow goodness-of-fit test *p* > 0.05 indicates no evidence of poor model fit.

The AUC represents apparent discrimination of the final multivariable model within the study dataset, as the ROC analysis was performed using predicted probabilities generated from the same dataset used to fit the model. No separate training and validation datasets were used, and no bootstrap resampling or cross-validation procedure was performed. Therefore, the reported AUC should be interpreted as an apparent, in-sample measure of discrimination rather than as an internally validated estimate of predictive performance. Formal internal validation, such as bootstrap resampling or cross-validation, and external validation in independent populations would be required to assess the stability and generalizability of the model's discriminatory performance. Multicollinearity among independent variables was examined using variance inflation factors (VIF) and tolerance statistics.

All statistical analyses were conducted using Stata version 17.0 (StataCorp, College Station, TX). A two-sided *p*-value < 0.05 was considered statistically significant. The regression analyses were conducted at the individual participant level, with categorical predictors represented using indicator (dummy) variables and the prespecified reference categories shown in Table 2.

## Results

The analysis included 2,236 adults aged 20 years and above residing in the five study villages. The mean age of participants was 41.1 ± 14.7 years. More than half of the participants were aged 30–59 years (57.8%), while 22.9% were aged 20–29 years and 19.3% were aged 60 years or older (Table 1). Men constituted a slightly higher proportion of the study population (53.5%) compared with women (46.5%). The majority of participants were married (80.9%), while 15.9% were unmarried and 3.2% were separated or widowed. Educational attainment in the study population was generally low. Nearly two-thirds of participants (63.1%) were illiterate, while 19.3% had completed primary education and 17.6% had attained secondary education or higher. Occupationally, laborers and farmers constituted the largest group (38.7%), followed by homemakers (33.8%). Smaller proportions of participants were unemployed (13.1%), students (5.1%), or engaged in other occupations (9.4%). The mean monthly household income was INR 3,122 (SD, 3,477).

Among the 2,236 adults included in the analysis, a total of 239 individuals reported current tobacco use, yielding an overall prevalence of 10.7%. Marked gender differences were observed in tobacco use prevalence. Tobacco use was substantially higher among men (17.9%) compared with women (2.4%), indicating strong gender disparities within the study population (Table 4). Age-related differences were also observed. Tobacco use prevalence increased across age groups, with the highest prevalence among adults aged ≥60 years (13.2%), followed by those aged 30–59 years (11.5%), while the lowest prevalence was observed among younger adults aged 20–29 years (6.4%).

**Table 4:** Bivariate association between sociodemographic, behavioral and exposure factors and current tobacco use (*N* = 2,236).

Variable	Tobacco user *n* (%)	Non-user *n* (%)	*p*-value†
Gender	
Male	214 (17.9)	983 (82.1)	< 0.001
Female	25 (2.4)	1,014 (97.6)	
Age group (years)	
20–29	33 (6.4)	479 (93.6)	0.001
30–59	149 (11.5)	1,144 (88.5)	
≥60	57 (13.2)	374 (86.8)	
Education level	
Illiterate	172 (12.2)	1,239 (87.8)	0.004
Primary	41 (9.5)	390 (90.5)	
Secondary†	26 (6.6)	368 (93.4)	
Occupation group	
Homemaker	13 (1.7)	743 (98.3)	< 0.001
Labor/Farmer	180 (18.6)	786 (81.4)	
Student	2 (1.8)	111 (98.2)	
Unemployed	18 (6.2)	274 (93.8)	
Other occupations^*^	26 (23.9)	83 (76.1)	
Alcohol consumption	
Yes	153 (83.6)	30 (16.4)	< 0.001
No	86 (4.2)	1,967 (95.8)	
Household smoking exposure	
Yes	74 (42.0)	102 (58.0)	< 0.001
No	165 (8.0)	1,895 (92.0)	
Workplace smoking exposure	
Yes	80 (52.6)	72 (47.4)	< 0.001
No	159 (7.6)	1,925 (92.4)	

†*p*-values were obtained using Pearson's chi-square test; ^*^Other occupations include business owners, drivers, private sector workers, vendors and skilled workers; categories with small cell sizes were merged to ensure statistical validity.

Table 4 presents the bivariate associations between socio-demographic, behavioral and environmental exposure variables and current tobacco use. Significant differences in tobacco use were observed across several socio-demographic factors. Tobacco use was more prevalent among men, older adults, and individuals with lower educational attainment. Occupational patterns were also evident. Tobacco use prevalence was highest among individuals engaged in labor or farming (18.6%) and those in other occupations (23.9%), whereas substantially lower prevalence was observed among homemakers (1.7%) and students (1.8%).

Alcohol consumption showed a strong association with current tobacco use (83.6% among alcohol users compared with 4.2% among non-users). Of the 183 participants who reported alcohol consumption, 153 (83.6%) were current tobacco users, whereas 30 (16.4%) were non-users. Among the 2,053 participants who did not report alcohol consumption, 86 (4.2%) were current tobacco users and 1,967 (95.8%) were non-users. This highly imbalanced distribution across the alcohol consumption and tobacco-use categories may contribute to the large magnitude of the observed association and warrants cautious interpretation. The large adjusted odds ratio should therefore be interpreted cautiously in the context of the observed distribution of the data, in which tobacco use was highly concentrated among participants reporting alcohol consumption. Because the multivariable model estimated the association using the binary alcohol-consumption variable while simultaneously adjusting for the prespecified sociodemographic, behavioral, and environmental covariates, the resulting adjusted odds ratio reflects the conditional association between alcohol consumption and tobacco use within this model rather than a causal effect.

Similarly, household smoking exposure and workplace smoking exposure were strongly associated with tobacco use. Tobacco use prevalence was 42.0% among individuals reporting household smoking exposure, compared with 8.0% among those without such exposure. Workplace smoking exposure also showed a strong association, with 52.6% of exposed individuals reporting tobacco use compared with 7.6% among those not exposed.

The results of the univariate and multivariable logistic regression analyses are presented in Table 5. In univariate analyses, male gender was associated with substantially higher odds of tobacco use compared with females (OR = 8.83, 95% CI: 5.78–13.49). After adjustment for age group, education, occupation, alcohol consumption, household smoking exposure and workplace smoking exposure, the association remained statistically significant but was attenuated (AOR = 2.12, 95% CI: 1.08–4.18). Age demonstrated a graded association with tobacco use. Compared with adults aged 20–29 years, individuals aged 60 years and above had significantly higher odds of tobacco use in both unadjusted (OR = 2.21, 95% CI: 1.41–3.47) and adjusted analyses (AOR = 3.42, 95% CI: 1.67–6.99). The association observed for adults aged 30–59 years in univariate analysis did not remain statistically significant after adjustment. Educational attainment showed an inverse relationship with tobacco use in univariate analysis; however, this association was no longer statistically significant after adjustment for other socio-demographic and behavioral variables. With respect to occupation, homemakers had significantly lower odds of tobacco use compared with individuals in other occupations (AOR = 0.22, 95% CI: 0.07–0.64). Other occupational categories were not statistically significantly associated with tobacco use after adjustment.

**Table 5:** Factors associated with tobacco use among adults (*N* = 2,236): Univariate and multivariable logistic regression analysis.

Variable	Crude OR (95% CI)	Adjusted OR†(95% CI)
Gender	
Female (ref)	1.00	1.00
Male	8.83 (5.78–13.49) ^***^	2.12 (1.08–4.18) ^*^
Age group (years)	
20–29 (ref)	1.00	1.00
30–59	1.89 (1.28–2.80) ^**^	1.66 (0.89–3.10)
≥60	2.21 (1.41–3.47) ^**^	3.42 (1.67–6.99) ^**^
Education level	
Illiterate (ref)	1.00	1.00
Primary	0.76 (0.53–1.09)	0.68 (0.39–1.18)
Secondary+	0.51 (0.33–0.78) ^**^	0.68 (0.35–1.31)
Occupation group	
Other occupations (ref)	1.00	1.00
Homemaker	0.06 (0.03–0.11) ^***^	0.22 (0.07–0.64) ^**^
Labor/Farmer	0.73 (0.46–1.17)	1.09 (0.52–2.27)
Student	0.06 (0.01–0.25) ^**^	0.47 (0.09–2.63)
Unemployed	0.21 (0.11–0.40) ^**^	0.40 (0.16–1.04)
Alcohol consumption	
No (ref)	1.00	1.00
Yes	116.65 (74.60–182.39) ^***^	64.39 (38.73–107.06) ^***^
Household smoking exposure	
No (ref)	1.00	1.00
Yes	8.33 (5.94–11.69) ^***^	1.40 (0.73–2.70)
Workplace smoking exposure	
No (ref)	1.00	1.00
Yes	13.45 (9.41–19.23) ^***^	3.19 (1.70–6.00) ^**^

^*^p < 0.05; ^**^ *p* < 0.01; ^***^ *p* < 0.001. Adjusted odds ratios were estimated using conventional multivariable binary logistic regression; household- and village-level clustering was not explicitly incorporated into the model.

Alcohol consumption demonstrated the strongest association with tobacco use in the regression analysis. Individuals reporting alcohol consumption had markedly higher odds of tobacco use in both unadjusted (OR = 116.65, 95% CI: 74.60–182.39) and adjusted models (AOR = 64.39, 95% CI: 38.73–107.06). The adjusted estimate was obtained from the prespecified multivariable binary logistic regression model, in which age group, gender, education level, occupation group, alcohol consumption, household smoking exposure, and workplace smoking exposure were entered simultaneously. In the underlying data, 153 of 183 participants who reported alcohol consumption (83.6%) were current tobacco users, compared with 86 of 2,053 participants who did not report alcohol consumption (4.2%). This highly imbalanced distribution of tobacco use across alcohol-consumption categories may contribute to the unusually large magnitude of the odds ratio and warrants cautious interpretation. The substantial co-occurrence of alcohol consumption and tobacco use may reflect shared behavioral patterns within the study population. Household smoking exposure and workplace smoking exposure also showed important associations with tobacco use. While household smoking exposure was significantly associated with tobacco use in univariate analysis, the association did not remain statistically significant after adjustment. In contrast, workplace smoking exposure remained statistically significantly associated with tobacco use after adjustment (AOR = 3.19, 95% CI: 1.70–6.00).

The multivariable logistic regression model demonstrated high apparent discriminatory performance within the study sample. Receiver operating characteristic (ROC) analysis yielded an area under the curve (AUC) of 0.926 (95% CI: 0.907–0.945) (Table 6; Figure 2). Because the ROC analysis was based on predicted probabilities generated from the same dataset used to fit the model, this AUC represents apparent in-sample discrimination and should not be interpreted as evidence of validated or generalizable predictive performance. No separate internal validation procedure, such as bootstrap resampling or cross-validation, was performed. The ROC analysis was therefore considered supplementary and exploratory rather than a validated prediction assessment.

**Table 6:** Discriminatory performance of the multivariable logistic regression model for tobacco use (*N* = 2,236).

Measure	Value
Area under the ROC curve (AUC)	0.926
Standard error	0.010
*p*-value (vs. AUC = 0.5)	< 0.001
95% Confidence interval	0.907–0.945
Positive cases (tobacco users)	239
Negative cases (non-users)	1,997

ROC analysis was performed using predicted probabilities from the fully adjusted logistic regression model. An AUC value closer to 1.0 indicates better model discrimination.

**Figure 2:**
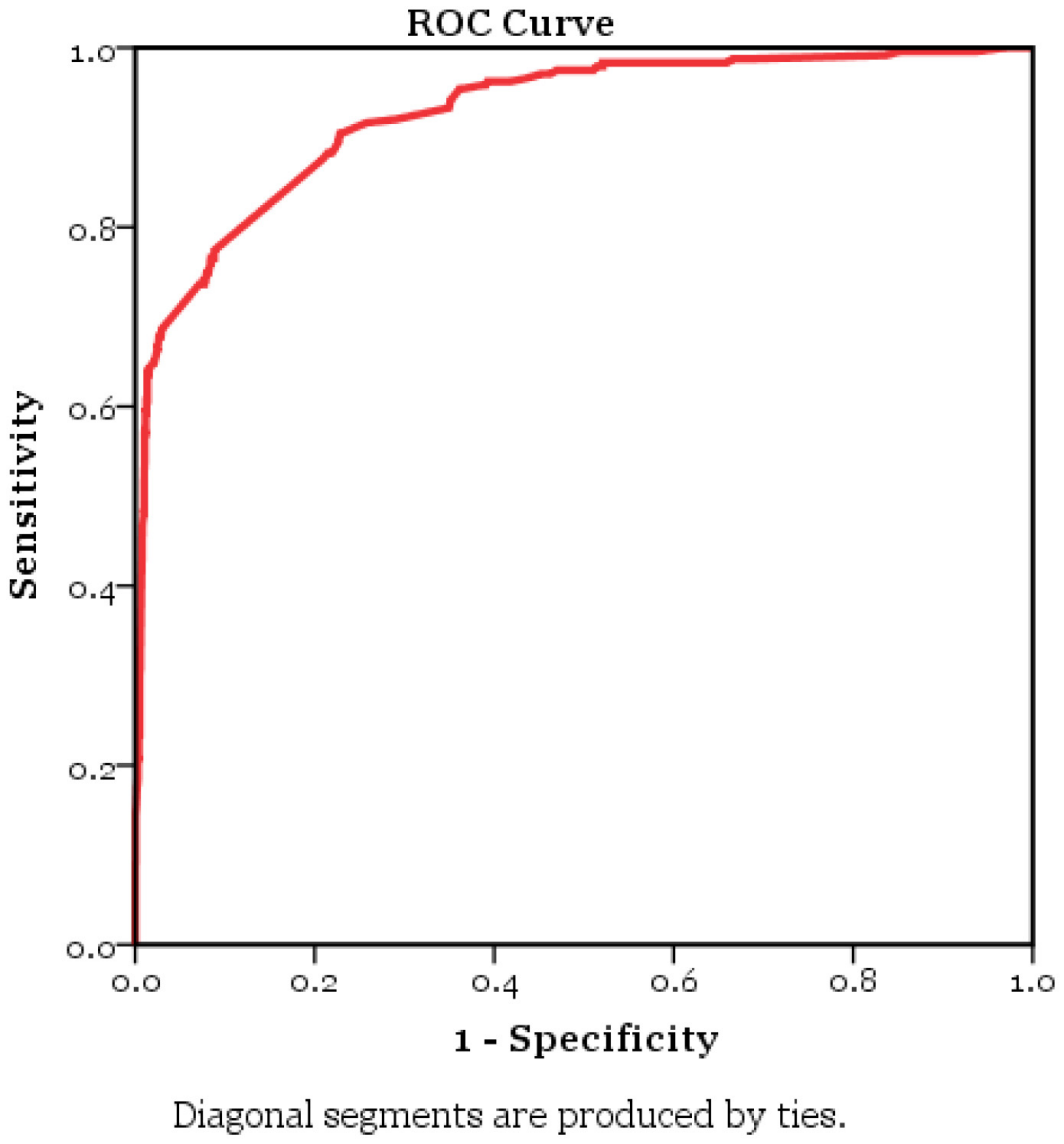
Receiver operating characteristic (ROC) curve showing the exploratory discriminatory performance of the fully adjusted multivariable logistic regression model for current tobacco use among adults.

Model calibration was acceptable, as indicated by the Hosmer–Lemeshow goodness-of-fit test (χ^2^ = 15.01, *p* = 0.059), suggesting adequate agreement between predicted and observed outcomes. For descriptive purposes, the Youden index identified an exploratory probability threshold of 0.053, corresponding to a sensitivity of 0.92 and specificity of 0.71 (Table 7). These measures describe the sensitivity–specificity trade-off observed within the study sample and were included as supplementary information on model discrimination. The probability threshold should not be interpreted as a clinically validated screening or prediction cut-off, and the sensitivity and specificity should not be interpreted as estimates of diagnostic accuracy in an independent population. Further formal internal validation, such as bootstrap resampling or cross-validation, and external validation in independent populations would be required before considering the model for predictive application.

**Table 7:** Exploratory sensitivity and specificity of the multivariable logistic regression model at the Youden-Index threshold.

Probability cut-off	Sensitivity	Specificity
0.053	0.92	0.71

The exploratory probability threshold was selected using the Youden index (sensitivity + specificity – 1) and is presented for descriptive assessment of model discrimination within the study sample.

## Discussion

This study examined the socio-demographic, behavioral and environmental correlates of current tobacco use among adults living in a socially and economically marginalized tribal population in northern India. The findings indicate that tobacco use was associated with a combination of individual, behavioral, occupational and environmental characteristics. These patterns are consistent with previous evidence showing that tobacco use may cluster within broader social and occupational contexts in socially disadvantaged populations (42).

The overall prevalence of current tobacco use in this study was 10.7%, which appears lower than the prevalence reported in several studies conducted among tribal populations in India. For example, a community-based study among persons of tribal origin in Wayanad, Kerala, reported a prevalence of current tobacco use of 39.1%, with smokeless tobacco accounting for the majority of reported use (43). Similarly, studies conducted among other tribal communities in India have reported substantially higher levels of tobacco use (44). The comparatively lower prevalence observed in the present study may reflect heterogeneity across tribal populations in terms of geographical location, sociocultural practices, patterns of tobacco consumption, availability and accessibility of tobacco products, and population composition. Differences in study design, age structure, operational definitions of current tobacco use, and survey period may also contribute to variation in prevalence estimates. Although the present study included both smoked and smokeless tobacco products, tobacco use was based on self-report, and under-reporting cannot be excluded, particularly where tobacco use may carry social stigma. Therefore, the lower prevalence observed in this study should be interpreted in the context of substantial heterogeneity in tobacco-use patterns across tribal communities rather than as evidence of uniformly lower tobacco use among tribal populations in northern India.

Tobacco use was substantially more common among men than women, consistent with national and regional evidence from South Asia (1, 7, 13, 14, 37). This gender difference may reflect differences in the social acceptability of tobacco use, occupational exposure and gender-specific behavioral norms. These findings support consideration of gender-specific social contexts when designing tobacco-control strategies.

Age-related differences in tobacco use were also observed in this study. Adults aged 60 years and above demonstrated significantly higher odds of tobacco use compared with younger adults. Similar age gradients have been reported in national surveys and regional studies across South Asia, where tobacco use may persist from earlier adulthood into later life, particularly among socially disadvantaged populations (16, 18, 29). This pattern supports the value of incorporating age-specific considerations into tobacco-control and cessation strategies.

Educational attainment showed an inverse association with tobacco use in univariate analyses but did not remain statistically significant after adjustment. This attenuation suggests that the observed association between education and tobacco use may partly reflect differences in socio-economic and occupational characteristics. Similarly, although tobacco use was more common among individuals in manual and informal occupations in bivariate analyses, most occupational categories were not statistically significantly associated with tobacco use after adjustment, except for the lower odds observed among homemakers. These findings are broadly consistent with previous evidence linking tobacco use with socio-economic and occupational context (21–23, 26–28).

One of the most notable findings of this study was the exceptionally strong association between alcohol consumption and tobacco use. Alcohol consumption was associated with substantially higher odds of tobacco use in both unadjusted and adjusted analyses, with an adjusted odds ratio of 64.39 (95% CI: 38.73–107.06). This unusually large estimate warrants cautious interpretation. In the underlying data, 153 of 183 participants who reported alcohol consumption (83.6%) were current tobacco users, whereas 86 of 2,053 participants who did not report alcohol consumption (4.2%) were tobacco users. Thus, tobacco use was highly concentrated among participants reporting alcohol consumption, resulting in a markedly imbalanced exposure–outcome distribution. Although these cell counts do not by themselves establish a sparse-data problem, such an imbalanced distribution may contribute to instability or inflation of a very large odds ratio and should therefore be considered when interpreting the magnitude of the estimate. The adjusted odds ratio should also be interpreted as a model-based conditional association, because the estimate was derived from a conventional multivariable logistic regression model that simultaneously adjusted for age group, gender, education level, occupation group, household smoking exposure, and workplace smoking exposure. In addition, tobacco and alcohol use frequently co-occur within shared social and occupational contexts, reflecting broader patterns of behavioral risk clustering (19, 29, 30, 32). The observed association may therefore reflect co-occurring behavioral patterns and shared contextual factors rather than a direct causal effect of alcohol consumption on tobacco use. Given the cross-sectional design, temporal ordering and causality cannot be established. The finding should consequently be interpreted as a strong statistical association and supports the consideration of co-occurring behavioral risks in tobacco-control strategies (36, 37).

Household smoking exposure and workplace smoking exposure were associated with tobacco use. While both household smoking exposure and workplace smoking exposure were associated with tobacco use in univariate analyses, only workplace smoking exposure remained statistically significantly associated after adjustment. This finding is consistent with evidence that tobacco use may cluster within occupational environments where smoking is socially common (8, 36, 38). In informal labor settings, workplace smoking may represent both exposure to environmental tobacco smoke and a marker of shared social and occupational contexts. However, given the cross-sectional design, the observed association should not be interpreted as evidence that workplace smoking exposure causes tobacco use.

Taken together, the findings indicate that tobacco use in this population was associated with multiple structural, behavioral and environmental characteristics. The observed pattern is consistent with the view that tobacco use occurs within broader socio-economic, occupational and behavioral contexts (45). These findings support consideration of context-specific tobacco-control approaches that address both individual and environmental factors, while recognizing that the cross-sectional design does not permit causal inference.

From a policy perspective, these findings highlight the need for context-specific tobacco-control strategies in socially marginalized populations. Integrating tobacco control with alcohol-related risk reduction strategies, strengthening smoke-free workplace environments in informal sectors, and addressing gender-specific social contexts may be relevant components of tobacco-control strategies in similar settings. Such approaches align with the broader objectives of the WHO Framework Convention on Tobacco Control and the Sustainable Development Goals, which emphasize reducing health inequalities and addressing the social determinants of non-communicable diseases.

### Limitations

Several limitations of this study should be considered when interpreting the findings. First, the cross-sectional design limits the ability to establish causal relationships or determine the temporal ordering of exposures and outcomes. Although strong associations were observed between tobacco use and factors such as alcohol consumption and workplace smoking exposure, the study design does not allow determination of whether these exposures preceded tobacco use or occurred concurrently. Reverse causality is therefore possible, particularly for behavioral factors that may co-occur over time. Longitudinal studies are needed to better understand temporal relationships and behavioral trajectories related to tobacco use across the life course.

Second, tobacco use, alcohol consumption, household smoking exposure, and workplace smoking exposure were assessed using self-reported measures, which may be subject to recall bias and social desirability bias. Under-reporting of tobacco use may be particularly relevant among women and socially marginalized populations in South Asia because of social stigma and cultural norms surrounding substance use (33, 46). Such misclassification may have resulted in underestimation of tobacco use prevalence and may have attenuated some associations.

Third, although the analysis incorporated several key socio-demographic, behavioral, and environmental variables, residual confounding cannot be excluded. Other potentially relevant factors associated with tobacco use, including peer networks, psychosocial stress, mental health status, social support structures, and access to cessation services, were not measured in the present study. These unmeasured factors may partly explain the observed associations.

Fourth, the exceptionally strong association observed between alcohol consumption and tobacco use should be interpreted with caution. The very large adjusted odds ratio may partly reflect the highly imbalanced distribution of tobacco use across alcohol-consumption categories, with tobacco use occurring in 83.6% of participants reporting alcohol consumption compared with 4.2% among those not reporting alcohol consumption. Although these cell counts do not conclusively demonstrate sparse-data bias, the concentration of tobacco-use events among alcohol users may contribute to an unusually large and potentially unstable odds ratio. In addition, alcohol consumption and tobacco use may co-occur within shared social and occupational contexts, and residual confounding by unmeasured behavioral or social factors may also contribute to the observed magnitude of association. Therefore, the estimate should be interpreted as a strong statistical association rather than as evidence of a causal effect.

Fifth, tobacco-use variables did not capture frequency, duration, or intensity of tobacco consumption, limiting the ability to distinguish between occasional and heavy users. This constraint restricts the assessment of dose–response relationships and cumulative exposure.

Sixth, the study population was drawn from a specific tribal community residing in five villages within a single geographic area. Although this focus provides valuable insight into an under-researched and socially marginalized population, the findings may not be fully generalizable to other tribal groups, rural populations, or urban settings in India. Nevertheless, the social patterning observed in this study is broadly consistent with findings from national surveys and regional studies.

Seventh, participants were recruited within households and villages, and individuals sharing the same household or village may have correlated characteristics because of common socioeconomic, cultural, behavioral, and environmental contexts. The primary analysis used conventional binary logistic regression at the individual participant level and did not explicitly incorporate household- or village-level random effects or cluster-adjusted standard errors. Consequently, residual clustering and its potential effects on the precision of the estimated associations cannot be excluded. This limitation should be considered when interpreting the reported odds ratios and confidence intervals. Future studies with a larger number of sampling clusters should consider multilevel or other cluster-aware analytical approaches to explicitly account for the hierarchical structure of the data.

## Conclusion

This study found that current tobacco use among adults in a socially marginalized tribal population was associated with gender, older age, alcohol consumption, occupational characteristics and workplace smoking exposure. The strong association observed between alcohol consumption and tobacco use and the association between workplace smoking exposure and tobacco use highlight the co-occurrence of behavioral and environmental risk factors in this population. However, because of the cross-sectional design, these findings should be interpreted as associations and should not be considered evidence of causal relationships. The findings may help inform context-specific tobacco-control strategies, including strengthening smoke-free workplace environments and integrating tobacco-control efforts with approaches addressing co-occurring behavioral risks in socially disadvantaged communities.

## Data Availability

The original contributions presented in the study are included in the article/supplementary material, further inquiries can be directed to the corresponding authors.

## References

[1] GBD 2019 Tobacco Collaborators. Spatial, temporal, and demographic patterns in prevalence of smoking tobacco use and attributable disease burden in 204 countries and territories, 1990-2019: a systematic analysis from the Global Burden of Disease Study 2019. Lancet. (2021) 397:2337–60. doi: 10.1016/S0140-6736(21)01169-7 34051883 PMC8223261

[2] Jha P Peto R. Global effects of smoking, of quitting, and of taxing tobacco. N Engl J Med. (2014) 370:60–8. doi: 10.1056/NEJMra1308383 24382066

[3] Murray CJ Lopez AD. Global mortality, disability, and the contribution of risk factors: Global Burden of Disease Study. Lancet Lond Engl. (1997) 349:1436–42. doi: 10.1016/S0140-6736(96)07495-8 9164317

[4] WHO. WHO Tobacco Trends Report: 1 in 5 Adults Still Addicted to Tobacco. (2025). Available online at: https://www.who.int/news/item/06-10-2025-who-tobacco-trends-report-1-in-5-adults-still-addicted-to-tobacco (Accessed December 24, 2025).

[5] Bilano V Gilmour S Moffiet T d'Espaignet ET Stevens GA Commar A . Global trends and projections for tobacco use, 1990-2025: an analysis of smoking indicators from the WHO comprehensive information systems for tobacco control. Lancet Lond Engl. (2015) 385:966–76. doi: 10.1016/S0140-6736(15)60264-1 25784347

[6] Ng M Freeman MK Fleming TD Robinson M Dwyer-Lindgren L Thomson B . Smoking prevalence and cigarette consumption in 187 countries, 1980-2012. JAMA. (2014) 311:183–92. doi: 10.1001/jama.2013.284692 24399557

[7] WHO. 2025 Global Progress Report on Implementation of the WHO Framework Convention on Tobacco Control. Geneva: World Health Organization (2025). Available online at: https://fctc.who.int/docs/librariesprovider12/meeting-reports/2025-global-progress-report-on-implementation-of-the-who-fctc.pdf?sfvrsn=8a0d2f8_1 (Accessed October 1, 2026).

[8] Chung-Hall J Craig L Gravely S Sansone N Fong GT. Impact of the WHO FCTC over the first decade: a global evidence review prepared for the impact assessment expert group. Tob Control. (2019) 28:s119–28. doi: 10.1136/tobaccocontrol-2018-054389 29880598 PMC6589489

[9] Kuthe SM Bandyopadhyay V Aparnavi P Venkatesh U Padhi BK Gandhi AP. Electronic cigarettes (ENDS OR ENNDS) smoking in Asian countries after complete vaping ban: a systematic review and meta-analysis. Subst Use Misuse. (2025) 61:793–802. doi: 10.1080/10826084.2025.2577277 41174357

[10] Gravely S Giovino GA Craig L Commar A D'Espaignet ET Schotte K . Implementation of key demand-reduction measures of the WHO Framework Convention on Tobacco Control and change in smoking prevalence in 126 countries: an association study. Lancet Public Health. (2017) 2:e166–74. doi: 10.1016/S2468-2667(17)30045-2 29253448

[11] Holford TR Meza R Warner KE Meernik C Jeon J Moolgavkar SH . Tobacco control and the reduction in smoking-related premature deaths in the United States, 1964-2012. JAMA. (2014) 311:164–71. doi: 10.1001/jama.2013.285112 24399555 PMC4056770

[12] Levy DT Tam J Kuo C Fong GT Chaloupka F. The impact of implementing tobacco control policies: the 2017 tobacco control policy scorecard. J Public Health Manag Pract. (2018) 24:448–57. doi: 10.1097/PHH.0000000000000780 29346189 PMC6050159

[13] Hitchman SC Fong GT. Gender empowerment and female-to-male smoking prevalence ratios. Bull World Health Organ. (2011) 89:195–202. doi: 10.2471/BLT.10.079905 21379415 PMC3044247

[14] Samet JM Yoon SY World Health Organization. Gender, Women, and the Tobacco Epidemic. Geneva: World Health Organization (2010). Available online at: https://www.who.int/publications/i/item/9789241563970 (Accessed December 24, 2025).

[15] Sinha DN Gupta PC Ray C Singh PK. Prevalence of smokeless tobacco use among adults in WHO South-East Asia. Indian J Cancer. (2012) 49:342–6. doi: 10.4103/0019-509X.107726 23442396

[16] Sreeramareddy CT Pradhan PMS Mir IA Sin S. Smoking and smokeless tobacco use in nine South and Southeast Asian countries: prevalence estimates and social determinants from demographic and health surveys. Popul Health Metr. (2014) 12:22. doi: 10.1186/s12963-014-0022-0 25183954 PMC4151025

[17] WHO SEARO. Report of the Expert Group Consultation on Smokeless Tobacco and Public Health in WHO-SEAR Countries. Geneva: World Health Organization, South-East Asia Regional Office (2015). Available online at: http://www.searo.who.int/tobacco/meetings/sea_tobacco_23_slt_mumbai_july_2015_report.pdf (Accessed October 1, 2026).

[18] Singh A Ladusingh L. Prevalence and determinants of tobacco use in India: evidence from recent Global Adult Tobacco Survey data. PLoS ONE. (2014) 9:e114073. doi: 10.1371/journal.pone.0114073 25474196 PMC4256395

[19] Palipudi KM Gupta PC Sinha DN Andes LJ Asma S McAfee T . Social determinants of health and tobacco use in thirteen low and middle income countries: evidence from Global Adult Tobacco Survey. PLoS ONE. (2012) 7:e33466. doi: 10.1371/journal.pone.0033466 22438937 PMC3306395

[20] Jha P Ramasundarahettige C Landsman V Rostron B Thun M Anderson RN . 21st-century hazards of smoking and benefits of cessation in the United States. N Engl J Med. (2013) 368:341–50. doi: 10.1056/NEJMsa1211128 23343063

[21] Sorensen G Gupta PC Pednekar MS. Social disparities in tobacco use in Mumbai, India: the roles of occupation, education, and gender. Am J Public Health. (2005) 95:1003–8. doi: 10.2105/AJPH.2004.045039 15914825 PMC1449300

[22] Jarvis M Wardle J. Social Patterning of Individual Health Behaviours: the Case of Cigarette Smoking (2009). Available online at: https://www.semanticscholar.org/paper/Social-patterning-of-individual-health-behaviours%3A-Jarvis-Wardle/fc7ed7ed8860a78500f8ae19661f3fc3283c5e3c (Accessed December 24, 2025).

[23] Lee AJ Crombie IK Smith WC Tunstall-Pedoe HD. Cigarette smoking and employment status. Soc Sci Med. (1991) 33:1309–12. doi: 10.1016/0277-9536(91)90080-V 1776044

[24] Venkatesh U Aparnavi P Mogan KA Durga R Pearson J Kishore S . Determinants of substance use among young people attending primary health centers in India. Glob Ment Health Camb Engl. (2024) 11:e23. doi: 10.1017/gmh.2024.13 38572250 PMC10988150

[25] Mogan KA Venkatesh U Kapoor R Kumar M. Determinants of substance use among young people attending an urban primary health center in Delhi. Int J Adolesc Med Health. (2020) 34. doi: 10.1515/ijamh-2019-0243 32474456

[26] Krieger N. Theories for social epidemiology in the 21st century: an ecosocial perspective. Int J Epidemiol. (2001) 30:668–77. doi: 10.1093/ije/30.4.668 11511581

[27] Berkman LF Kawachi I. “A historical framework for social epidemiology”. In:Berkman LF Kawachi I Glymour MM, editors. Social Epidemiology. New York: Oxford University Press (2014). doi: 10.1093/med/9780195377903.003.0001

[28] Marmot M. Social determinants of health inequalities. Lancet Lond Engl. (2005) 365:1099–104. doi: 10.1016/S0140-6736(05)71146-6 15781105

[29] Sreeramareddy CT Ramakrishnareddy N Harsha Kumar H Sathian B Arokiasamy JT. Prevalence, distribution and correlates of tobacco smoking and chewing in Nepal: a secondary data analysis of Nepal Demographic and Health Survey-2006. Subst Abuse Treat Prev Policy. (2011) 6:33. doi: 10.1186/1747-597X-6-33 22185233 PMC3266635

[30] Khanal V Adhikari M Karki S. Social determinants of tobacco consumption among Nepalese men: findings from Nepal Demographic and Health Survey 2011. Harm Reduct J. (2013) 10:40. doi: 10.1186/1477-7517-10-40 24359118 PMC3880042

[31] Pradhan PMS Niraula SR Ghimire A Singh SB Pokharel PK. Tobacco use and associated factors among adolescent students in Dharan, Eastern Nepal: a cross-sectional questionnaire survey. BMJ Open. (2013) 3:e002123. doi: 10.1136/bmjopen-2012-002123 23418297 PMC3585970

[32] Commission on Social Determinants of Health. Closing the Gap in a Generation: Health Equity through Action on the Social Determinants of Health. Geneva: World Health Organization (2008). Available online at: https://www.who.int/publications/i/item/9789241563703 (Accessed October 1, 2026).

[33] Graham H. Women's smoking and family health. Soc Sci Med. (1987) 25:47–56. doi: 10.1016/0277-9536(87)90206-1 3616697

[34] Venkatesh U Ahmed A Grover A Joshi A Bera OP Dixit AM . Uncovering mental well-being profiles in urban slums of Gorakhpur, India: a cluster-based approach using SWEMWBS. Glob Ment Health. (2026) 13:e18. doi: 10.1017/gmh.2026.10132 41695255 PMC12893872

[35] Venkatesh U Rahaman M Ahmed A Joshi HS Dixit AM Grover A . Household-level WASH conditions and waterborne diseases among the Vantangiya scheduled tribe in India: a micro-scale spatial analysis. Sci Rep. (2026) 16:21407. doi: 10.1038/s41598-026-50775-w 42204183 PMC13350806

[36] Okechukwu C Davison K Emmons K. “Changing health behaviors in a social context”. In:Berkman LF Kawachi I Glymour MM, editors. Social Epidemiology. New York: Oxford University Press (2014). p. 365–95. doi: 10.1093/med/9780195377903.003.0010

[37] Morrow M Barraclough S. Gender equity and tobacco control: bringing masculinity into focus. Glob Health Promot. (2010) 17:21–8. doi: 10.1177/1757975909358349 20595351

[38] WHO. WHO Framework Convention on Tobacco Control (WHO FCTC) (2005). Available online at: https://fctc.who.int/convention/9241591013 (Accessed December 24, 2025).

[39] NCD Countdown 2030 Collaborators. NCD countdown 2030: pathways to achieving sustainable development goal target 3.4. Lancet. (2020) 396:918–34. doi: 10.1016/S0140-6736(20)31761-X 32891217 PMC7470795

[40] Venkatesh U Santhosh VN Joshi HS Grover A Bera OP Mohanraj PS . Evaluating cognitive impairment among a geriatric population in India using the Indian Council of Medical Research (ICMR)–multilingual dementia research and assessment (MUDRA) toolbox. Indian J Psychiatry. (2025) 67:959–68. doi: 10.4103/indianjpsychiatry_424_25 41211500 PMC12594067

[41] Venkatesh U Ahmed A. Statistical data types and visualisation techniques in clinical and public health research: a methodological framework and practical guide. NMO J. (2026) 20:201–7. doi: 10.4103/JNMO.JNMO_17_26

[42] Ahmed A Mohammadi A Bergquist R Moghabeli R. Subnational inequalities and spatially varying associations of elevated blood glucose among women in India: a national district-level biomarkers analysis. BMC Public Health. (2026) 26:1890. doi: 10.1186/s12889-026-27482-2 42057028 PMC13274304

[43] Javed R Dinesh A Aswathy S Vasudevan S Mathew M Reynold S. Prevalence, pattern and determinants of tobacco use among persons of tribal origin in a primary care centre in Wayanad, Kerala. Int J Community Med Public Health. (2020) 7:3153. doi: 10.18203/2394-6040.ijcmph20203393

[44] Sadath A Jose K Jiji K Mercy V Ragesh G Arensman E. Prevalence and determinants of substance use among indigenous tribes in South India: findings from a tribal household survey. J Racial Ethn Health Disparities. (2022) 9:356–66. doi: 10.1007/s40615-021-00964-2 33495925

[45] Mohammadi A Ahmed A Pishgar E Fatima M Bergquist R. The geography of water pipe use: a case study in Tabriz City, Northwestern Iran. ISPRS Int J Geo-Inf. (2026) 15:169. doi: 10.3390/ijgi15040169

[46] Jung-Choi KH Khang YH Cho HJ. Hidden female smokers in Asia: a comparison of self-reported with cotinine-verified smoking prevalence rates in representative national data from an Asian population. Tob Control. (2012) 21:536–42. doi: 10.1136/tobaccocontrol-2011-050012 21972062

